# Analysis of the *Citrullus colocynthis* Transcriptome during Water Deficit Stress

**DOI:** 10.1371/journal.pone.0104657

**Published:** 2014-08-13

**Authors:** Zhuoyu Wang, Hongtao Hu, Leslie R. Goertzen, J. Scott McElroy, Fenny Dane

**Affiliations:** 1 Department of Horticulture, Auburn University, Alabama, United States of America; 2 Department of Biological Sciences, Auburn University, Alabama, United States of America; 3 Department of Crop, Soil and Environmental Sciences, Auburn University, Alabama, United States of America; University of Delhi South Campus, India

## Abstract

*Citrullus colocynthis* is a very drought tolerant species, closely related to watermelon (*C. lanatus* var. *lanatus*), an economically important cucurbit crop. Drought is a threat to plant growth and development, and the discovery of drought inducible genes with various functions is of great importance. We used high throughput mRNA Illumina sequencing technology and bioinformatic strategies to analyze the *C. colocynthis* leaf transcriptome under drought treatment. Leaf samples at four different time points (0, 24, 36, or 48 hours of withholding water) were used for RNA extraction and Illumina sequencing. qRT-PCR of several drought responsive genes was performed to confirm the accuracy of RNA sequencing. Leaf transcriptome analysis provided the first glimpse of the drought responsive transcriptome of this unique cucurbit species. A total of 5038 full-length cDNAs were detected, with 2545 genes showing significant changes during drought stress. Principle component analysis indicated that drought was the major contributing factor regulating transcriptome changes. Up regulation of many transcription factors, stress signaling factors, detoxification genes, and genes involved in phytohormone signaling and citrulline metabolism occurred under the water deficit conditions. The *C. colocynthis* transcriptome data highlight the activation of a large set of drought related genes in this species, thus providing a valuable resource for future functional analysis of candidate genes in defense of drought stress.

## Introduction

Water is essential for plant growth in modern agriculture [1]. Drought delays the development of crops, and strongly affects morphology, as well as physiological processes like transpiration, photosynthesis, respiration and translocation of assimilates [2]. Drought avoidance can be achieved through morphological changes in plants, such as decreased stomatal conductance, reduced leaf area, and extensive root systems [3]. Drought tolerance is achieved by physiological and molecular mechanisms, including osmotic adjustment, antioxidant and scavenger compounds [4]. Both strategies involve the induction of specific genes and proteins, such as dehydrins (dehydration-induced proteins), key enzymes for osmolyte biosynthesis, and detoxification enzymes [5], [6].

Plant species have developed diverse strategies to adapt and thrive in all kinds of climates and terrains to deal with extreme changes in the environment. These strategies are supported by rich and complex metabolic networks that enable the plant to synthesize a wide range of compounds. Plant responses to abiotic stresses involve interactions and crosstalk between many molecular pathways. High throughput screening techniques such as transcriptome sequencing have been used to study the adaptability of plants to drought [7]. This led to the discovery of many drought related genes. For example, PIP aquaporins were found to fine-tune the environment in response to declining water availability [8]. However, few natural allelic variants have been cloned for drought related traits, so QTL, RNA sequencing, and other trait isolation methods are needed to improve methodology for exploring complex multivariate data [9], [10].

The cucurbit family is a large family with several economically important species, such as watermelon (*Citrullus lanatus*), melon (*Cucumis melo*), cucumber (*Cucumis sativus*) and several *Cucurbita* species with edible fruits [11]. *Citrullus colocynthis* (L.) Schrad (2n = 2x = 22), the bitter apple, closely related to domesticated watermelon (*Citrullus lanatus* var. *lanatus*), is a very drought-tolerant perennial herbaceous species in the Cucurbitaceae family [12]. It can survive arid environments by maintaining its water content under severe stress conditions. *C. colocynthis* is an important medicinal plant and a source of valuable oil [12]. Its seeds were found in several early Egyptian, Libyan and Near Eastern sites from about 4000 BC. This species grows in sandy areas throughout northern Africa, southwestern Asia and the Mediterranean region [11], [12]. The species has been used as a model to elucidate the function of genes implicated in the stress response ultimately leading to enhancement of stress tolerance in cucurbit crops through genetic manipulation. Si et al. [13] found dynamic gene expression changes in *C. colocynthis* root tissues using cDNA amplified fragment length polymorphism (cDNA-AFLP) technique.

Several research groups have used next generation sequencing technologies to study gene expression profiles in species of the cucurbit family. Guo et al [14] used 454 sequencing technology to study the comprehensive profile for watermelon fruit flesh tissues, Grassi et al [15] studied carotenoid pathway regulators in ripening watermelon fruit. The draft genome of watermelon (*C. lanatus*, 2n = 2x = 22, ∼425 Mb) was analyzed by Guo et al [16] using three different watermelon subspecies. Comparative genomic analysis provided an evolutionary scenario for the origin of the 11 watermelon chromosomes derived from a 7-chromosome paleohexaploid eudicot ancestor. The genome sequence of cucumber (*Cucumis sativus*, 2n = 2x = 14) has been completed, and the genome of melon (*Cucumis melo*, 2n = 2x = 24) is being sequenced under the Spanish Genomics Initiative (MELONOMICS) [17], [18]. Liu et al [19] used sequencing techniques to identify conserved and novel miRNA in watermelon, while Wincker [20] used comparative analysis of genomes between watermelon and sweet orange to detect the traits related to their domestication.

Here, high-throughput sequencing of the leaf transcriptome from *C. colocynthis* provides a glimpse at drought related genes in this uniquely drought tolerant cucurbit species. The lack of extensive genomic and functional genomic resources in *Citrullus* species has hampered research and breeding of the cultivated and economically important *C. lanatus*. This study should facilitate the identification of valuable multiple genes, needed for complex interactions of plant species with the environment.

## Materials and Methods

### Plant Materials and RNA Extraction

*C. colocynthis* seedlings were grown in Sunshine Mix #8 under a 16 h light/8 h dark photoperiod at 26°C day, 22°C night temperature. Seedlings with 2–3 true leaves (2–3 week old) in 50 ml containers were exposed to drought by withholding water for 0 (day 1, D1), 24 (day 2, D2), 36 (day 3, D3) or 48 hours (day 4, D4). True leaf samples were collected each day at noon, flash frozen in liquid nitrogen and stored at −80°C. RNA was subsequently extracted using the TRIzol method [21].

### Preparation of cDNA Library and Sequencing

Illumina sequencing was performed at the HudsonAlpha Institute of Biotechnology (Huntsville, AL) following manufacturer's instructions. RNA-Seq reads were first processed to remove rRNA sequence contamination. First strand cDNA was synthesized with reverse transcriptase and random primers using the small fragment RNAs as template. Second strand cDNA was then synthesized followed by phosphorylation by T4 DNA polymerase. The cDNA fragments were 3′ adenylated and ligated to the Illumina's paired-end adapters. Enrichment of cDNA templates was conducted following fifteen cycles of PCR amplification. In total, over 20*4Mp were sequenced for mapping assembly and differential expression analysis. Raw sequence data are available for download at NCBI Sequence Read Archive (SAMN02769576, SAMN02769577, SAMN02769578, and SAMN02769579).

### Assembly

Raw sequencing data were filtered Z (0.05) using the CLC Genomics workbench (CLC Bio, Aarhus, Denmark). Paired–end sequences from the four samples were used to construct the *de novo C. colocynthis* transcriptome assembly with default parameters. Assembly reads were also assembled against the watermelon (*C. lanatus*) genome sequences, which were downloaded from the Cucurbit Database (http://www.icugi.org/cgi-bin/ICuGI/index.cgi). Reads were filtered and assembled using the CLC genomics workbench. The parameters used were as follows: 2 points of mismatch cost, 2 points of insertion cost, 2 points of deletion cost, 0.5 as length fraction, 0.95 as similarity fraction. After the *de novo* assembly and watermelon mapping assembly, we used Trinity to assemble all the contigs with the default parameters.

### Identification of Full Length cDNAs

Two methods were used to identify full length cDNAs. First, BLASTX searching (E value: 1e^−10^) was used to detect the matched cDNAs in SwissProt database; second, ESTScan 2.0 was used to identify the translated sequences. Sequences with either start codon (ATG) and stop codon (TAG/TGA/TAA), or sequences with start codon (ATG) and homologue to a known protein with ≥80% similarity, were chosen as full length cDNAs.

### Expression Analysis Using Transcriptome Reference

Pair-end sequencing reads of the four libraries were filtered using CLC Genomics workbench (0.05) before mapping to the references sequences from assembled cDNAs. First, read counts of each unigene were converted to reads per million (RPM). Secondly, statistical analysis using Kal's test [22] provided in CLC Genomics workbench (P<0.05 and fold change ≥1.5) was conducted. These transcripts were annotated against the reference sequences.

### Gene Ontology Analysis

The functional annotation software BLAST2GO (http://www.blast2go.com/b2ghome) was used to conduct gene ontology (GO) analysis of *C. colocynthis* genes in this study. The databases used were SwissProt and NCBI. BLAST E-value was set at 1.0e^−3^. The major GO analysis was determined by BLAST, mapping, and annotation. Results are presented as a bar chart showing the percent of genes belonging to each group.

### qRT-PCR Analysis

For cDNA synthesis, 500 ng of the total RNA for each sample (the same RNA was used for RNA-seq analysis) was used in reverse transcription with ProtoScript First Strand cDNA synthesis kit from New England BioLabs (Ipswich, MA). qRT-PCR was performed with SYBR-Green Supermix from Bio-Rad (Hercules, CA) in an Eppendorf Mastercycler ep realplex (Hauppauge, NY). Table S1 contains gene specific primer sequences. Each reaction contained 10 µl of SYBR-Green supermix, 1 µl of cDNA template, 1 µl forward primer (4 µm), 1 µl reverse primer (4 µm), and 7 µl ddH_2_O. The qRT-PCR program consisted of one cycle at 95°C for 15 sec, followed 40 cycles of 15 sec at 95°C, 15 sec at 55°C, and 30 sec at 72°C. The relative expression data was compared with actin [13] from *C. colocynthis*. Quantification of the relative transcript levels was performed using the comparative C_T_ method. The induction ratio (IR) was calculated as recommended by the manufacturer and corresponds to 2^−ΔΔCT^, where ΔΔCT =  (C_T, target gene_, -C_T, actin_)_treatment_- (C_T, target_-C_T, actin_)_control_. All experiments were replicated three times.

## Results and Discussion

### Transcriptome Assembly Results

The transcriptome of *C. colocynthis* leaves following 4 days of drought stress was assembled and assessed following paired-end (2*50 bp) Illumina sequencing. The Illumina platform yielded an average of 24 million high-quality reads per sample (Table 1). All sample reads were used to construct a *de novo* assembly. A reference assembly was constructed using the completely sequenced watermelon genome. A total of 20,581 contigs were generated (Table 2). The contigs had an average length of 1350 bp and N50 of 1870 bp. BLASTX was used against SwissProt and ESTscan of translated protein sequences to detect full length cDNAs. A total of 5,038 full length cDNAs were detected in our sequencing assembly (Table 2).

**Table 1:** Paired end read statistics before and after trimming of the *Citrullus colocynthis* transcriptome at 4 time points.

Time point	Day 1		Day 2		Day 3		Day 4	
	Before	After	Before	After	Before	After	Before	After
Reads	**21,194,778**	**20,339,776**	**23,566,720**	**22,626,577**	**23,435,694**	**22,531,826**	**27,388,920**	**26,288,785**
	**21,379,596**	**20,537,526**	**23,774,222**	**22,846,047**	**23,626,206**	**22,738,550**	**27,737,584**	**26,649,390**
Average	**21,287,187**	**20,438,651**	**23,670,471**	**22,736,312**	**23,530,950**	**22,635,188**	**27,563,252**	**26,469,088**
% Trimmed	**96.15**		**96.11**		**96.19**		**96.03**	

**Table 2:** Summary details of sequences produced after assembly of *Citrullus colocynthis* reads.

	Length/Number of reads
N50	**1870 bp**
Average length	**1350 bp**
Minimum length	**201 bp**
Maximum length	**1956 bp**
Total number of contigs	**20581**
Full length cDNAs	**5038**

**Figure 1:**
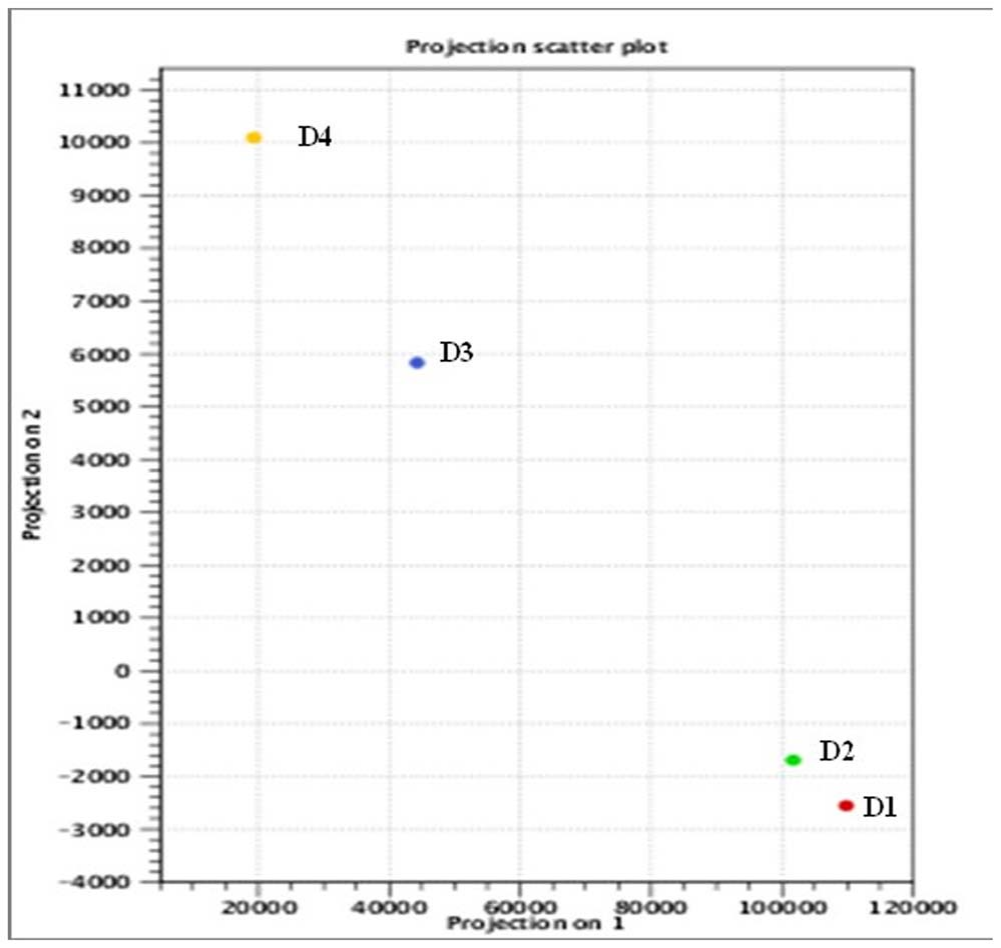
Principal component analysis of the leaf transcriptome of *Citrullus colocynthis* at 0 (D1), 24 (D2), 36 (D3) and 48 (D4) hours of withholding water. PCA analysis was conducted using the CLC workbench. The X-axis indicates principal component 1, the Y-axis principal component 2. Data from D1 and D2 are more closely related than data from D3 and D4.

Principle component analysis (PCA) was implemented in the CLC workbench. The results (Figure 1) illustrate differential gene expression patterns in *C. colocynthis* seedlings following four days of withholding water. Gene expression patterns on D1 and D2 were nearly similar, while results from D3 and D4 showed substantial differences as compared to D1 and D2, and gene expression patterns detected on D4 were very different from all other time points as a result of drought stress.

### Differentially Expressed Genes Involved in Response to Drought Stress in *C. colocynthis*

The paired-end reads were mapped to the reference genomes after filtration. Read counts of each unigene were converted to reads per million (RPM). The read number of each cDNA was divided by the total number of reads per day (1, 2, 3, or 4) from the data set, and multiplied by 10^6^. Statistical analysis was conducted using Kal's test (p<0.05 and fold change ≥1.5). Genes showing non-significant and significant changes in read counts are shown in Table S2. Each sample was compared to the day 1 sample reads for analysis of their significance level of gene expression. The read results indicated that 59 genes showed significant changes at D2, D3, and D4 as compared to D1; 13 genes showed significant differential expression at D2 and D3 as compared to D1; 30 genes showed significant differential expression early, at D2, as compared to D1; 897 genes showed changes at D3 and D4 as compared to D1; 341 genes were only differentially expressed at D3; 1191 genes were regulated late, at D4 only, under drought stress. In conclusion, the *C. colocynthis* gene expression patterns showed dramatic changes with 2545 genes showing significant changes, mostly occurring late under drought conditions (D3 and D4 of withholding water).

**Figure 2:**
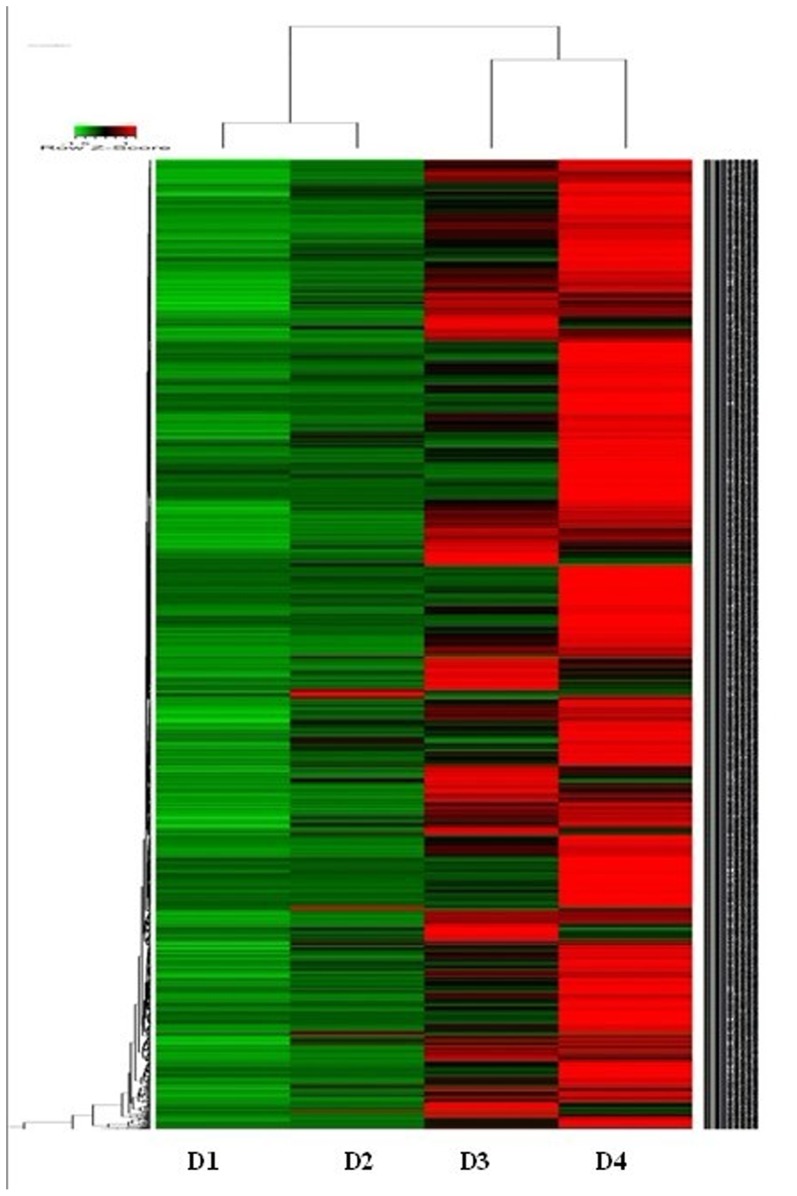
Heat map of the leaf transcriptome of *Citrullus colocynthis* depicting changes in transcript patterns of 2545 gene transcript clusters under water deficit treatment. Heatmap was produced using R. Each red or green block represents normalized expression value of a gene detected at 0 (D1), 24 (D2), 36 (D3) and 48 (D4) hours of withholding water. Red indicates higher gene expression values across treatment, while green indicates lower expression values across treatment.

The heat map depicted in Figure 2 corresponded to the principal component analysis. D3 and D4 transcripts were clustered together, and D1 and D2 transcripts were clustered. Also significant changes were seen at D3 and D4 suggesting that the transcriptional response of many genes was up-regulated during drought. Strong effects were especially observed on D3 and D4.

### Gene Ontology (GO) Classification

**Figure 3:**
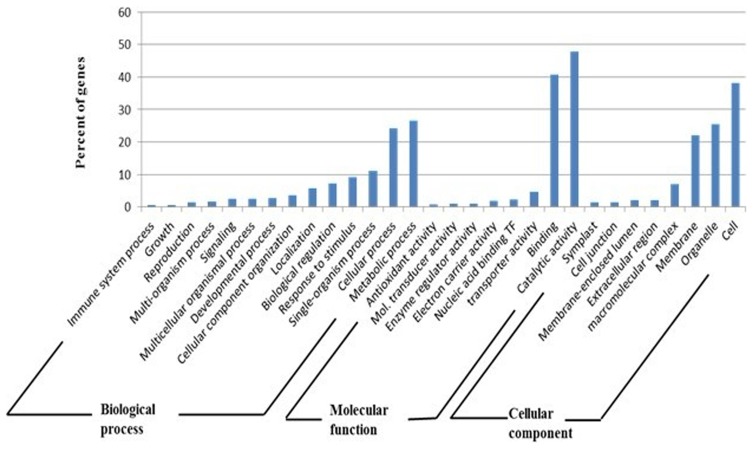
Gene Ontology (GO) of significantly changed *Citrullus colocynthis* genes under drought stress. Based on high-score BLASTX matches in the NR (non-redundant) plant protein database, *C. colocynthis* genes were classified into three main GO categories. The y-axis indicates the percentage of a specific category of genes in each main category.

To functionally categorize significantly changed genes in *C. colocynthis* under drought treatment, gene ontology analysis by BLAST2GO was performed. *C. colocynthis* unigenes were categorized in three main GO categorizes: biological process (2672), molecular function (1368) and cellular component (1053). These GO terms were further divided into several sub-categories (Figure 3). In the biological process category, single organism process genes accounted for more than 20% of the biological process genes. In the molecular function category, more than 40% of genes were associated with a catalytic activity. In the cellular category, more than 35% of the genes were associated with the cellular component.

### Validation of Illumina Expression Patterns by qRT-PCR Analysis

**Figure 4:**
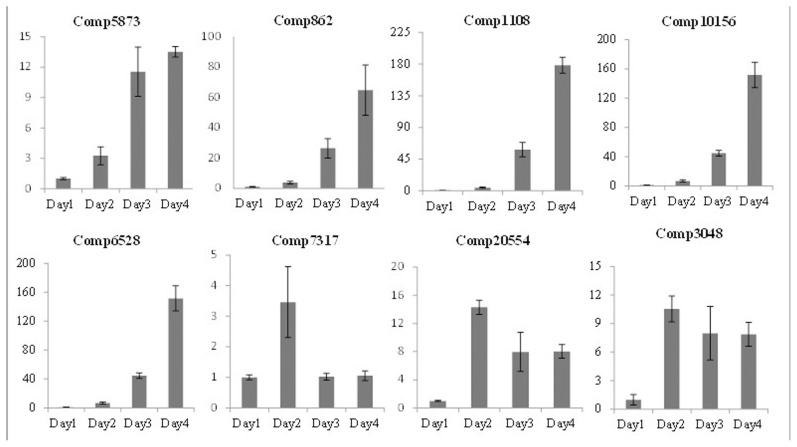
qRT-PCR analysis of 8 selected *Citrullus colocynthis* genes under drought treatment. The y-axis indicates relative expression compared with Day 1 expression of each gene. The x-axis shows days of water-withholding time points.

**Figure 5:**
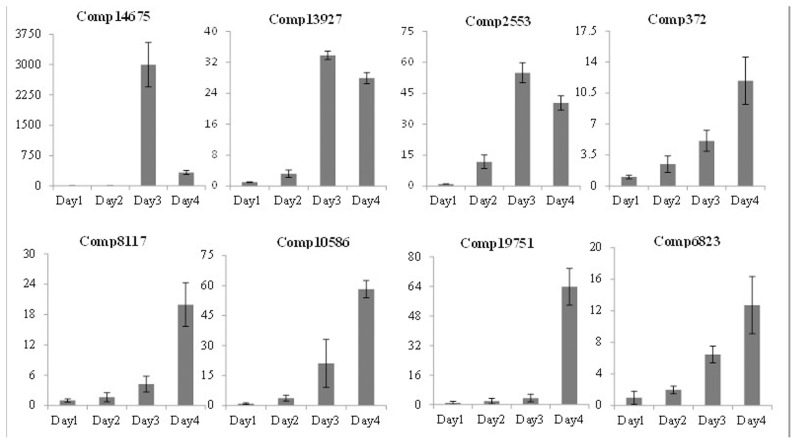
qRT-PCR analysis of 8 additional *Citrullus colocynthis* genes under drought treatment. The y-axis indicates relative expression compared with Day 1 expression of each gene. The x-axis shows days of water-withholding time points.

To confirm the reliability of the Illumina sequencing read analysis, 8 candidate genes were selected and their expression was compared at D4 and D1 using qRT-PCR. The expression patterns resulting from qRT-PCR showed general agreement with those from the Illumina sequencing analysis (Table 3). Discrepancies with respect to ratio of fold changes between sequencing and qRT-PCR analysis can be attributed to the essentially different algorithm and sensitivity of the two techniques [23]. In the deep-sequencing method the absolute expression rather than relative expression as in qRT-PCR analysis is used. Transcriptional qRT-PCR analyses of 16 genes during the drought treatments are shown in Figures 4 and 5.

**Table 3:** Validation of the RNA-Seq expression profiles of selected *C. colocynthis* genes by qRT-PCR.

Gene ID	Hit ID	Annotations	Log2FC*-RNA seq	Log2 FC-qRT-PCR
Comp14675	Cla000300	Heat shock protein	7.60	8.37
Comp13927	Cla001351	Cold shock protein	3.41	4.8
Comp2553	Cla002576	MA3 domain-containing protein	2.15	5.33
Comp372	Cla002814	Bax inhibitor-1	1.90	3.56
Comp8117	Cla012143	Drought stress related gene	2.87	4.31
Comp10586	Cla013687	MYB	4.09	5.86
Comp19751	Cla022362	WRKY	6.27	6.00
Comp6823	Cla022169	MADS	2.07	3.67

Transcripts identified as D4 significantly changed genes were compared to transcripts at D1. The table shows the log2 fold change calculated from D4 expression vs D1 expression for RNAseq and qRT-PCR analysis. FC = fold change. Gene ID is gene named in *C. colocynthis*, and Hit ID is blastx of gene with watermelon ID number.

Gene Comp5873 is homeobox-leucine zipper protein, with significant upregulation during all days of drought in *C. colocynthis*. It is known that the expression of several homeobox-leucine zipper proteins is correlated to stress. Athb-12, a homeobox-leucine zipper domain protein from *Arabidopsis*, is functionally involved in salt tolerance in yeast [24]. Hahb-4, a homeobox-leucine zipper gene is potentially involved in water stress in sunflower [25]. Comp862 belongs to the glutathione S-transferase (GST) family, which contains heterogeneous, multifunctional dimeric proteins. This gene is highly up-regulated (60x) during drought in *C. colocynthis*. It is thought that GSTs are involved in cellular detoxification [26]. Comp1108 is a member of the NAC (NAM/ATAF1, 2/CUC2) gene family and NACs are known to be involved in numerous biological processes, including drought stress [27].

Comp10156 is a member of the GID1 (GIBBERELLIN INSENSITIVE DWARF1) family in *C. colocynthis* with high expression under drought conditions. GID1 is a soluble GA receptor in rice [28]. GA-GID1 complex interacts with DELLA proteins, which are negative regulators of GA signaling pathway. RD22, a known dehydration responsive gene in *Arabidopsis*, which is mediated by abscisic acid (ABA), may have physiological and molecular significance for processes underlying memory functions of plants in response to ABA and light pulses [29], [30]. One RD22-like protein from soybean can alleviate salinity and osmotic stress [31]. RD22-like genes (Comp 6528) with significant up-regulation (>160x) under drought in *C. colocynthis* were confirmed by qRT-PCR, especially after days of withholding water.

NDR1 (NON-RACE-SPECIFIC DISEASE RESISTANCE1) plays an important role in coordinating broader cellular processes in response to stress and bacterial pathogen infection [32]. The chemical β-aminobutyric acid, which is known to induce resistance in plants, primed the expression of many genes, and NDR1/NHL10 was one of them [33]. Comp7317 gene, which is a member of NDR1/NH10 showed significant changes at D2 only during the early stage of water deficit stress.

GRAS (for GA Insensitive, REPRESSOR of gal-3 (RGA), SCARECROW (SCR)) transcription factors, have a major function in plant development and environmental adaption. These TFs are particularly implicated in the modulation of plant tolerance to stressors as cold, drought, salinity by crosstalks via GA to ABA-dependent and ABA-independent pathways [34]. For example, SCL7 confers salt and drought tolerance in *Arabidopsis* [35]. SCL14 is involved in the detoxification of xenobiotics and possibly endogenous harmful metabolites [36]. Comp20554, which belongs to the GRAS transcription factor family, showed significant up-regulation especially on D2.

The expression profile of Comp3048, which codes for a methyltransferase, maintained high levels during drought stress, especially on D2. It was found that myo-inositol-O-methyltransferase (Imt1) responded to low temperature stress in transgenic *Arabidopsis* [37]. Trithorax-like H3K4 methyltransferase from barley is drought inducible [38]. The methylation of myo-inositol catalyzed by myo-inositol methyltransferase (IMT) occurs when plants are under abiotic stress. Over-expression of IMT resulted in improved tolerance to dehydration and salt stress treatment in *Arabidopsis* [39].

Heat-shock proteins (HSPs) are environmentally induced proteins that enable plants to cope with heat and other environmental stresses. For example, *Trichoderma harzianum* Hsp70 transgenic *Arabidopsis* is abiotic stress tolerant [40]. Similarly HSP22 was found to be highly upregulated in *C. colocynthis* roots during drought conditions [13]. Overexpression of GmHsp90S can decrease damage of abiotic stresses in *Arabidopsis* [41]. Comp14675 belongs to the HSP family, and showed up-regulation at later stages of D3 and D4 (Figure 5).

Plant cold shock proteins (CSP) are very conserved among various plant genera [42]. The first CSP identified was WCSP1 from winter wheat, which did accumulate in response to low-temperature stress [43]. Similarly in *C. colocynthis* CSP (Comp13927) was up-regulated during later stages of water deficit.

Comp2553, one MA3 domain-containing protein gene, showed marked changes on D3 and D4 under drought conditions (Figure 5). Loss-of-function of ECIP1 (one MA3 domain-containing protein) resulted in enhanced ethylene response but altered salt response [44] in *Arabidopsis*.

Overexpression of plant BL-1 in *Arabidopsis* resulted in the attenuation of cell death induced by biotic stresses (pathogens) and abiotic stresses such as heat, cold, drought, salt and chemical-induced oxidative stresses [45]. BL-1 might function to control the level of the “pro-survival and pro-death” signals under multiple stress conditions in plants [46]. For example, cucumber BAX inhibitor-1 is a conserved cell death suppressor induced by cold stress and a negative regulator of programmed cell death (PCD) [47]. Comp372 encodes one BL-1 gene, which was up-regulated by drought at later stages (D3 and D4).

Comp8117, which is a homologue of a *Populus* EST (CU233481.1), is a drought stress related gene, up-regulated at D4. Comp10586, which encodes a member of the MYB transcription factor family, is up-regulated during the late stage of drought stress. Some MYB members have been shown to regulate plant responses to biotic and abiotic stress conditions [48]. For example, AtMYB96 acts through the ABA signaling pathway to induce pathogen resistance by promoting salicylic acid biosynthesis, and thus regulating stomata movement, drought tolerance and disease resistance in *Arabidopsis* [49], [50]. MYB88 might function directly or indirectly, as positive regulator of stress-responsive genes [51]. TaMYB30-B from wheat did improve drought stress tolerance in transgenic *Arabidopsis* [52]. Another family of transcription factors, the WRKY family, named for the WRKY domain of about 60 amino acids, contains a highly conserved WRKYGQK heptapeptide at its N-terminus and a zinc-finger-like motif at its C-terminus [53]. WRKY transcription factors are involved in multiple aspects of plant growth, development and stress [54]. Several TaWRKY in wheat with roles in the abiotic stress response acted in an ABA-dependent manner [55]. Here, gene Comp19751encodes a WRKY gene, which showed remarkable up-regulation at D4.

MADS-box family members function in reproductive development and stress [56]. For example, OsMADS25 and OsMADS27 transcripts accumulate in response to osmotic stress [57]. Comp6823, which encodes for a MADS-box gene in *C. colocynthis*, is up-regulated markedly at the last stage of drought (D4; Figure 5).

### Analysis of the Drought Stress Signaling Transcriptome in *C. colocynthis*

Drought stress signal transduction consists of several pathways including ionic and osmotic homeostasis signaling, detoxification response and growth regulation pathways [58]. Genes detected in *C. colocynthis* leaves during water deficit are listed in Table 4.

**Table 4:** List and number of several drought stress signaling pathway members detected in the *Citrullus colocynthis* transcriptome.

Ion Signaling	NO.
Calcium Binding Protein	10
**Protein Kinase Pathways For Osmotic Signaling**	
MAP kinase	16
**Osmotic Stress-Activated Phospholipid Signaling**	
PLC-like Phosphodiesterase	9
DAG	8
PIP2-like Aquaporin	5
**Detoxification Signaling**	
Heavy Metal Transport/Detoxification Protein	32
**Transcription Regulators**	
**Regulatory Proteins**	
MYB	131
MYC	43
NAC	42
Leucine-rich Repeat Proteins	150
**Functional Proteins**	
Heat Shock Protein 70	70
Heat Shock Protein 22	1
grpE Like Protein	4
RBOHD (respiratory burst oxidase)	2
VIP2 (VIRE2-INTERACTING PROTEIN2)	1
ABATransporter-like Protein	100
Synaptobrevin-related protein	5
Toc34-1 (Translocon outer envelope of chloroplast)	1
Beta-amylase	9
Puruvate Kinase	11
TIP1 (TIP GROWTH DEFECTIVE 1)	1
RD22	2

In the ion signaling pathway, calcium binding proteins are well known for their involvement in both biotic and abiotic stress response pathways [59]. The calcium ion (Ca^2+^) as a secondary messenger in plants is sensed by calmodulins (CaMs)/CaM-like protein (CMLs), the caldineurin B-like proteins (CBLs) and Ca2+-dependent protein kinases (CDPKs). CaM binds to CaM-binding proteins (CBPs), which function in different pathways under biotic and biotic stresses [60]. A total of 10 Ca^2+^ binding proteins were detected in the *C. colocynthis* transcriptome.

Protein phosphorylation is a central theme in the cell's response to stress. The MAP kinase cascade in transcript levels consist of a number of protein kinases, such as two-component histidine kinase, MAPKKK, MAPKK, MAPK etc. [61]. Here we detected 16 MAP kinases in the *C. colocynthis* transcriptome.

Membrane phospholipids can activate several types of phospholipases that cleave certain phospholipids to generate lipid messengers (eg. PA, DAG, IP3), which further regulate stress tolerance through modulation of stress-responsive gene expression [62]. Several members in this pathway, such as phospholipase C (PLC), diacylglycerol (DAG) and phosphotidylinositol 4,5-bisphosphate (PIP2)-like aquaporin were detected (Table 4).

Detoxification signaling can ameliorate the damage in plants under stresses [63], as noted in many other plant species. A total of 32 detoxification proteins were detected in the *C. colocynthis* transcriptome.

Molecular mechanisms regulating gene expression in response to drought stress have been studied by analyzing the functional transcription factors in ABA-dependent and ABA-independent pathways [6]. Several regulatory proteins in ABA-dependent or –independent pathway were detected, among which NAC, MYB/C and leucine-rich repeat proteins (LRR). These regulatory proteins can further modulate many responsive transcription factors. Several functional proteins such as heat shock protein (HSP) 70, HSP22, grpE like protein, respiratory burst oxidase D (RBOHD), VIRE2-Interacting protein2 (VIP2), ABA transporter-like protein, synaptobrevin-related protein, translocon outer envelope of chloroplast (Toc34-1), beta-amylase, TIP1 (TIP GROWTH DEFECTIVE 1), and RD22 were detected.

### Analysis of Phytohormone Signaling Mediators in *C. colocynthis*

Phytohormones play important roles in regulating plant responses under biotic and abiotic stress. Elaborate phytohormone signaling networks mediate the adaptability of plants to different environmental conditions [64]. Many phytohormones such as ABA, salicylic acid (SA), jasmonic acid (JA), auxin, ethylene and gibberellic acid (GA) are being studied for their role in abiotic stress responses [33], [65], [66]. It is known that downstream signaling proteins for auxin, GA, JA, and ABA are subjected to ubiquitin-dependent degradation [67]. Putative phytohormone signaling genes detected in *C. colocynthis* during the drought response are listed in Table 5.

**Table 5:** Overview of *Citrullus colocynthis* genes involved in phytohormone signaling.

**Function**	**Ethylene Pathway Major Members**	**Number**
Ethylene-insensitive protein	EIN	5
Ethylene receptors	ETR	3
Constitutive triple response proteins	CTR	1
EIN3-like (EIL) transcription factors	EIL	1
Ethylene response factors	ERF	118
**Function**	**Auxin Pathway Major Members**	**Number**
Receptors/F-box proteins	TIR	1
Ubiquitin ligase component	SCF	5
Target proteins	Aux/IAA	30
Auxin response factors	ARF	30
Auxin transport protein	PIN	6
**Function**	**JA Pathway Major Members**	**Number**
Receptor/F-box proteins	COI1	1
Target proteins	JAZ	1
JAZ interacted proteins	NINJA	4
Activator transcription factors	R2R3-MYB transcription factor	3
Activator transcription factors	MYC2,3,4	9
**Function**	**SA Pathway Major Members**	**Number**
Regulatory proteins	NPR1	2
SA mainly induced genes	WRKY	46
SA mainly induced genes	TGA2,3,5,6	6
**Function**	**ABA Pathway Major Members**	**Number**
ABA receptors	PYR/PYL/RCAR	19
PYR/PYL/RCAR interacted proteins	PP2C	3
Serine/threonine-protein kinase	SnRK2	1
SnRK2 target	ABI5	1
**Function**	**GA Pathway Major Members**	**Number**
GA receptors	GID1A/B/C/-like	11
E3 ubiquitin ligase	SLY1/SNZ	2
DELLA proteins	GA1, RGA, RGL1, 2, 3	4

Ethylene signaling pathway components were ordered into a hypothetical linear pathway based on both genetic (epistasis) analysis and biochemical interactions [68]. Almost all of the ethylene signaling homologous members (118 ethylene response factors or ERFs) were detected in the *C. colocynthis* transcriptome. APETALA2/ethylene responsive factor (AP2/ERF) transcription factors are well-known for mediating stress responses and development in plants [69].

The auxin response factor (ARF) family contains transcription factors that bind to auxin-responsive elements (AREs) in the promoters of primary auxin-responsive genes. Aux/IAAs are early auxin-response proteins that bind ARFs, therefore inhibiting ARE-mediated gene transcription. Aux/IAAs are involved in ubiquitin-mediated degradation, which is catalyzed by SCF E3 ubiquitin ligase. TIR1 can stimulate Aux/IAA proteolysis by binding auxin to this protein [70]. All of the major auxin signaling related transcription factors found in the *C. colocynthis* transcriptome are shown in Table 5. Similar to the auxin pathway, a novel family of transcriptional regulators, the jasmonate ZIM-domain (JAZ) proteins play a target part as Aux/IAA. In addition, COI1 plays a similar role as TIR1, while MYC and R2R3-MYB transcription factors work as ARFs [71]. Several JA signaling pathway related genes exist in the *C. colocynthis* transcriptome.

In the SA pathway, NPR1 (non-specific disease resistance 1) is a key regulator in SA-dependent defense signaling [72]. Similarly WRKY and TGA play major roles as transcriptional regulators in the SA pathway. We detected 2 NPR1 related proteins, 46 WRKYs and 6 TGAs in *C. colocynthis*, which might function in the *C. colocynthis* SA pathway.

Drought triggers the production of the phytohormone ABA, which in turn causes stomatal closure and induces expression of stress-related genes. The soluble PYR/PYL/RCAR receptors function at the apex of a negative regulatory pathway to directly regulate PP2C phosphatases, which in turn directly regulate SnRK2 kinases [73]. Several of the core transcription factors in the ABA pathway are listed in Table 5.

We also detected 11 GA receptor GIBBERELLIN INSENSITIVE DWARF1 (GID1), 4 DELLA growth inhibitors (DELLAs) and 2 F-box proteins (SLY1) and SNEEZY (SNZ), which play important roles in GA signaling pathways [74], [75].

### Cellular Metabolism under Drought Stress in *C. colocynthis*

Global gene expression analyses have shown substantial down-regulation of many photosynthetic genes under drought not only in *Arabidopsis* [76], but also several other species such as indica rice [77], [78]. Similarly, many photosystem I and II, chlorophyll a, b binding protein, and oxygen evolving enhancer protein genes [79], showed down-regulation during water deficit stress in *C. colocynthis* (Table 6).

**Table 6:** Photosynthesis-related and chlorophyll-related gene expression profiles.

Gene ID	Hit ID	Annotations	D1RPM	D2RPM	D3RPM	D4RPM
Comp808	Cla002545	Photosystem I psaA	1180	1066.9	513.6↓	418.8↓
Comp2604	Cla021635	Photosystem I subunit II	309	319	114↓	22.8↓
Comp9446	Cla002576	Photosystem I subunit III	470	428↓	97↓	39.1↓
Comp4444	Cla012670	Photosystem I subunit IV	274	219↓	70.4↓	8.3↓
Comp4514	Cla007871	Photosystem I subunit IV A	104.9	98.1↓	41.2↓	9.9↓
Comp2126	Cla004483	Photosystem I subunit V	292.9	281.8↓	95.6↓	23.5↓
Comp4727	Cla007940	Photosystem I subunit XI	257.2	229.5↓	61.4↓	9.6↓
Comp7482	Cla009814	Photosystem I subunit X	225.3	194.1↓	60.3↓	12.3↓
Comp5307	Cla011174	Photosystem I subunit X	199	176↓	44↓	4.6↓
Comp1999	Cla005420	Photosystem II polypeptide	225	194.1↓	60.3↓	12.3↓
Comp2011	Cla008554	Photosystem II 5 kDa protein	138.9	118.7↓	45.4↓	2.5↓
comp7744	Cla013942	Photosystem II Protein	495.8	515.8	331.4↓	116.7↓
Comp8614	Cla014815	Photosystem II reaction center W protein	310.2	265.5	117.3↓	28↓
Comp2016	Cla022723	Photosystem II core complex proteins psbY	78	76.3↓	24.7↓	7.7↓
Comp12757	Cla011748	Chlorophyll a–b binding protein 13	290.5	246.2↓	3.1↓	0.3↓
Comp6677	Cla013483	Chlorophyll a–b binding protein 3C	130.6	89.5↓	7.9↓	3.8↓
Comp1476	Cla013826	Chlorophyll a–b binding protein	671.4	583.9↓	42.9↓	8.8↓
Comp7589	Cla015680	Chlorophyll a–b binding protein 37	737.2	489.3↓	16.6↓	10.5↓
Comp2286	Cla017325	Chlorophyll a–b binding protein 3C	577.4	230.8↓	4.1↓	1↓
Comp544	Cla017983	Chlorophyll a–b binding protein 6	235.2	219.1↓	72.3↓	48.2↓
Comp3797	Cla018117	Chlorophyll a–b binding protein 6	632.9	502.3↓	154.8↓	38.3↓
Comp15099	Cla019595	Chlorophyll a–b binding protein 21	228.8	126.3↓	0.1↓	1
Comp1922	Cla001764	Chlorophyll a–b binding protein 8	609.1	470↓	241.6↓	58.6↓
Comp1584	Cla012368	Chlorophyll a–b binding protein 8	1082.2	884.8↓	111.1↓	281.1↓
Comp940	Cla009752	Chlorophyll a–b binding protein 21	652.6	514.8↓	43.3↓	3.6↓
Comp13569	Cla009753	Chlorophyll a–b binding protein 21	827.5	676.3↓	68.1↓	5.8↓
Comp4958	Cla022963	Chlorophyll a–b binding protein 7	538.2	404.7↓	110.1↓	18.8↓
Comp4043	Cla001790	Oxygen-evolving enhancer protein 1 of photosystem II	1088.6	795.9↓	334.6↓	82.8↓
Comp3077	Cla005429	Oxygen-evolving enhancer protein 2, chloroplastic	604.2	531.1↓	395.2↓	122.6↓
Comp5901	Cla019423	Oxygen-evolving enhancer protein 3	545.6	414.8↓	86.7↓	17↓

D1RPM-D4RMP are gene expression reads per millions for each day. ↓ indicates the down-regulation of each gene at each time point.

Citrulline, a non-protein amino acid intermediate in the arginine biosynthetic pathway, has been found to accumulate in leaves of drought tolerant watermelon under water deficit conditions [80], [81], [82]. Thus several factors related to the response of this model drought tolerant species to stress have been identified (Table 7, Figure S1). Citrulline metabolic genes (carbamoyl-phosphate synthetase, acetyl glutamate synthase, acetylornithine aminotransferase, aminoglutamate decarboxylase, acetylornithine deacetylase, and glutamate dehydrogenase) were found to be significantly up-regulated during drought.

**Table 7:** **Expression profiles of some citrulline metabolic genes during drought stress.**

Gene ID	Hit ID	Annotations	D1RPM	D2RPM	D3RPM	D4RPM
Comp1556	Cla006970	Carbamoyl-phosphate synthetase	64.6	70.9↑	159↑	175.8↑
Comp1556	Cla005591	Carbamoyl-phosphate synthetase	196.1	162.5	846.5↑	1542↑
Comp3843	Cla014787	Carbamoyl-phosphate synthetase	15	20.5↑	49.8↑	63.3↑
Comp11009	Cla016474	Proline dehydrogenase	2.2	3.9↑	11.6↑	31.4↑
Comp01186	Cla023055	Arinosuccinase	5.6	6.2	3.7	5.3
Comp5521	Cla020781	Orinithine carbamoyltransfrase	25.2	19.1↓	52.5↑	32.5
Comp6244	Cla015337	Acetylornithine aminotransferase	39	45.3↑	127↑	147.6↑
Comp6587	Cla008748	Glutamine amidotransferase	25.6	19.7	12.4	13.8
Comp15261	Cla017928	Glutamate 5-kinase	4.3	5.7	1.7	1.6
Comp3961	Cla019569	Orinithine-oxo-acid transaminase	19.3	30.5	28.1	17.1
Comp7707	Cla023055	Argininosuccinate lyase	5.6	6.2	3.7	5.3
Comp3468	Cla003592	Argininosuccinate lyase	22.9	26.8	35.4	25.6
Comp776_seq2	Cla002611	Arginosuccinate synthase	12.5	25.4	6	7.7
Comp776_seq1	Cla002609	Arginosuccinate synthase	18.2	18.9	2.4↓	2↓
Comp1556	Cla022915	Carbamoyl-phosphate synthetase	115.4	103.2	53.4↓	42↓

D1RPM-D4RMP indicate gene expression reads per millions for each day. ↑ and ↓ indicate the significant up-regulation and down-regulation of each gene at each time point.

One of the major research goals is to understand the molecular mechanisms underlying drought tolerance in plants. It is clear that drought triggers a wide variety of responses in *C. colocynthis*. Down regulation of many photosynthetic genes was observed especially at the later stages of drought. Up regulation of many transcription factors, stress signaling factors, detoxification genes, and genes involved in phytohormone signaling occurred throughout the water deficit experiment. Systematic approaches using genomic analyses should lead to the discovery of additional stress factors and provide us with a better understanding of the stress tolerance mechanism of this drought tolerant plant species.

## Supporting Information

Figure S1 **Citrulline metabolism pathway in** ***C. colocynthis*** **.**
(TIF)

Table S1 **qRT-PCR primer sequence information.**
(DOCX)

Table S2 **Non-significant and significantly changed** ***C. colocynthis*** **genes expressed as read counts during drought.**
(XLSX)

## References

[1] Guo Y, Tan J (2013) A biophotonic sensing method for plant drought stress. Sensor Actuat B-Chem 188: 519–524.

[2] Do PT, Degenkolbe T, Erban A, Heyer AG, Kopka J, et al (2013) Dissecting rice polyamine metabolism under controlled long-term drought stress. PLOS ONE 8: e60325. 23577102 10.1371/journal.pone.0060325 PMC3620119

[3] Levitt J (1980) Responses of plants to environmental stress: chilling, freezing and high temperature stresses, 2nd Ed New York: Academic Press.

[4] Bartels D, Sunkars R (2005) Drought and salt tolerance in plants. Cr Rev Plant Sci 24: 23–58.

[5] Reddy AR, Chaitanya KV, Vivekanandan M (2004) Drought induced responses of photosynthesis and antioxidant metabolism in higher plants. Plant Physiol 161: 1189–1202. 10.1016/j.jplph.2004.01.013 15602811

[6] Yamaguchi-Shinozaki K, Shinozaki K (2006) Transcriptional regulatory networks in cellular response and tolerance to dehydration and cold stresses. Annu Rev Plant Biol 57: 781–803. 16669782 10.1146/annurev.arplant.57.032905.105444

[7] Zhao J, Gao Y, Zhang Z, Chen T, Guo W, et al (2013) A receptor-like kinase gene (GbRLK) from *Gossypium barbadense* enhances salinity and drought-stress tolerance in *Arabidopsis* . BMC Plant Biol 13: 110–125. 23915077 10.1186/1471-2229-13-110 PMC3750506

[8] Sŭrbanovski N, Sargent DJ, Else MA, Simpson DW, Zhang H, et al (2013) Expression of *Fragaria vesca* PIP aquaporins in response to drought stress: PIP down-regulation correlates with the decline in substrate moisture content. PLOS ONE 8: e74945. 24086403 10.1371/journal.pone.0074945 PMC3781111

[9] Caccamo M, Grotewold E (2013) Turning over a new leaf in plant genomics. Genome Biol 14: 403. 23805981 10.1186/gb-2013-14-6-403 PMC3706968

[10] Juenger TE (2013) Natural variation and genetic constraints on drought tolerance. Curr Opin Plant Biol 16: 274–281. 23462639 10.1016/j.pbi.2013.02.001

[11] Jeffrey C (2008) A review of Cucurbitaceae. Bot J Linn Soc 81: 233–24.

[12] Dane F, Liu J, Zhang C (2006) Phylogeography of the bitter apple, *Citrullus colocynthis* . Genet Resour Crop Ev 54: 327–336.

[13] Si Y, Zhang C, Meng S, Dane F (2009) Gene expression changes in response to drought stress in *Citrullus colocynthis* . Plant Cell Rep 28: 997–1009. 19415285 10.1007/s00299-009-0703-5

[14] Guo S, Liu J, Zheng Y, Huang M, Zhang H, et al (2011) Characterization of the transcriptome dynamics during watermelon fruit development: sequencing, assembly, annotation and gene expression profiles. BMC Genomics 12: 454. 21936920 10.1186/1471-2164-12-454 PMC3197533

[15] Grassi S, Piro G, Lee JM, Zheng Y, Fei Z, et al (2013) Comparative genomics reveals candidate carotenoid pathway regulators of ripening watermelon fruit. BMC Genomics 14: 781. 24219562 10.1186/1471-2164-14-781 PMC3840736

[16] Guo S, Zhang J, Sun H, Salse J, William JL, et al (2013) The draft genome of watermelon (*Citrullus lanatus*) and resequencing of 20 diverse accessions. Nat Genet 45: 51–58. 23179023 10.1038/ng.2470

[17] Gonzalez VM, Garcia-Mas J, Arus P, Puigdomenech P (2010) Generation of a BAC-based physical map of the melon genome. BMC Genomics 11: 339. 20509895 10.1186/1471-2164-11-339 PMC2894041

[18] Huang S, Li R, Zhang Z, Li L, Gu X, et al (2009) The genome of the cucumber, *Cucumis sativus* L. Nat Genet 41: 1275–1281. 19881527 10.1038/ng.475

[19] Liu N, Yang J, Gao S, Xu Y, Zhang M (2013) Genome-wide identification and comparative analysis of conserved and novel microRNAs in grafted watermelon by high-throughput sequencing. PLOS ONE 8: e57359. 23468976 10.1371/journal.pone.0057359 PMC3582568

[20] Wincker P (2013) Genomics and fruit crop selection. Nature Genetics 45: 9–10. 23268131 10.1038/ng.2498

[21] Chomczynski P, Sacchi N (1987) Signal-step method of RNA isolation by acid guanidinium thiocyanate–phenol-chloroform extraction. Anal Biochem 162: 156–159. 2440339 10.1006/abio.1987.9999

[22] Kal AJ, van Zonneveld AJ, Benes V, van den Berg M, Koerkamp MG, et al (1999) Dynamics of gene expression revealed by comparison of serial analysis of gene expression transcript profiles from yeast grown on two different carbon sources. Mol Biol Cell 10: 1859–1872. 10359602 10.1091/mbc.10.6.1859 PMC25383

[23] Shi T, Gao Z, Wang L, Zhang Z, Zhuang W, et al (2012) Identification of differentially expressed genes associated with pistil abortion in Japanese apricot by genome-wide transcriptional analysis. Plos ONE 7: e47810. 23091648 10.1371/journal.pone.0047810 PMC3472986

[24] Shin D, Koo YD, Lee J, Lee H, Baek D, et al (2004) Athb-12, a homeobox-leucine zipper domain protein from *Arabidopsis thaliana*, increases salt tolerance in yeast by regulating sodium exclusion. Biochem Biophys Res 323: 534–540. 10.1016/j.bbrc.2004.08.127 15369784

[25] Gago GM, Almoguera C, Jordano J, Gonzalez DH, Chan RL (2002) Hahb-4, a homeobox-leucine zipper gene potentially involved in abscisic acid-dependent responses to water stress in sunflower. Plant Cell Environ 25: 633–640.

[26] Park H, Choung Y (2010) Evaluation of the biodegradation feasibility of antibiotics by three bacteria involving glutathione S-transferases. Can J Civ Eng 37: 814–819.

[27] Shaminuzzaman M, Vodkin L (2013) Genome-wide identification of binding sites for NAC and YABBY transcription factors and co-regulated genes during soybean seedling development by ChIP-Seq and RNA-Seq. BMC Genomics 14: 477. 23865409 10.1186/1471-2164-14-477 PMC3720225

[28] Ueguchi-Tanaka M, Ashikari M, Nakajima M, Itoh M, Katoh E, et al (2005) GIBBERELLIN INSENSITIVE DWARFS encodes a soluble receptor for gibberellin. Nature 437: 693–69. 16193045 10.1038/nature04028

[29] Goh CH, Nam HG, Park YS (2003) Stress memory in plants: a negative regulation of stomatal response and transient induction of rd22 gene to light in abscisic acid-entrained *Arabidopsis* plants. Plant J 36: 240–255. 14535888 10.1046/j.1365-313x.2003.01872.x

[30] Xu YH, Liu R, Yan L, Liu ZQ, Jiang SC, et al (2012) Light-harvesting chlorophyll a/b-binding proteins are required for stomatal response to abscisic acid in *Arabidopsis* . J Exp Bot 63: 1095–1106. 22143917 10.1093/jxb/err315 PMC3276081

[31] Wang H, Zhou L, Fu Y, Cheung M, Wong F, et al (2012) Expression of an apoplast-localized BURP-domain protein from soybean (GmRD22) enhances tolerance towards abiotic stress. Plant Cell Environ 35: 1932–1947. 22548236 10.1111/j.1365-3040.2012.02526.x

[32] Knepper C, Savory EA, Brad D (2011) *Arabidopsis* NDR1 is an integrin-like protein with a role in fluid loss and plasma membrane-cell wall adhesion. Plant Physiol 156: 286–300. 21398259 10.1104/pp.110.169656 PMC3091050

[33] Chen P, Parshant S, Laurent Z (2011) Priming of the *Arabidopsis* pattern-triggered immunity response upon infection by necrotrophic *Pectobacterium carotovorum* bacteria. Mol Plant Pathol 14: 58–70. 10.1111/j.1364-3703.2012.00827.x PMC6638802 22947164

[34] Golldack D, Li C, Mohan H, Probst N (2013) Gibberelins and abscisic acid signal crosstalk: living and developing under unfavorable conditions. Plant Cell Rep 32: 1007–1016. 23525744 10.1007/s00299-013-1409-2

[35] Ma H, Liang D, Shuai P, Xia X, Yin W (2010) The salt- and drought-inducible poplar GRAS protein SCL7 confers salt and drought tolerance in *Arabidopsis thaliana* . J Exp Bot 61: 4011–4019. 20616154 10.1093/jxb/erq217 PMC2935874

[36] Fode B, Siemsen T, Thurow C, Weigel R, Gatz C (2008) The *Arabidopsis* GRAS protein SCL14 interacts with Class II TGA transcription factors and is essential for the activation of stress-inducible promoters. Plant Cell 20: 3122–3135. 18984675 10.1105/tpc.108.058974 PMC2613660

[37] Zhu B, Peng RH, Xiong AS, Xu J, Fu XY, et al (2012) Transformation with a gene for myo-inositol O-methyltransferase enhances the cold tolerance of *Arabidopsis thaliana* . Biol Plantarum 56: 135–139.

[38] Papaefthimiou D, Tsataris AS (2012) Characterization of a drought inducible trithorax-like H3K4 methytransferase from barley. Biol Plantarum 56: 683–692.

[39] Ahn C, Park U, Park PB (2011) Increased salt and drought tolerance by D-ononitol production in transgenic *Arabidopsis thaliana* . Biochem Biophys Res 415: 669–674. 10.1016/j.bbrc.2011.10.134 22079289

[40] Montero-Barrientos M, Hermosa R, Cardoza RE, Gutiérrez S, Nicolás C, et al (2010) Transgenic expression of the *Trichoderma harzianum* hsp70 gene increases *Arabidopsis* resistance to heat and other abiotic stresses. Plant Physiol 167: 659–665. 10.1016/j.jplph.2009.11.012 20080316

[41] Xu J, Xue C, Xue D, Zhao J, Gai J, et al (2013) Overexpression of GmHsp90s, a heat shock protein (Hsp90) gene family form soybean, decreases damage of abiotic stresses in *Arabidopsis thaliana* . PLOS ONE 8: e69810. 23936107 10.1371/journal.pone.0069810 PMC3723656

[42] Karlson D, Imai R (2003) Conservative of the cold shock domain protein family in plants. Plant Physiol 131: 12–15. 12529510 10.1104/pp.014472 PMC1540277

[43] Karlson D, Nakaminami K, Toyomasu T, Imai R (2002) A cold-regulated nucleic acid-binding protein of winter wheat shares a domain with bacterial cold shock proteins. J Biol Chem 277: 35248–35256. 12122010 10.1074/jbc.M205774200

[44] Lei G, Shen M, Li Z, Zhang B, Duan K, et al (2011) EIN2 regulates salt stress response and interacts with a MA3 domain-containing protein ECIP1 in *Arabidopsis* . Plant Cell Environ 34: 1678–1692. 21631530 10.1111/j.1365-3040.2011.02363.x

[45] Ishikawa T, Watanabe N, Nagano N, Kawai-Yamada M, Lam E (2011) Bax inhibitor-1: a highly conserved endoplasmic reticulum-resident cell death suppressor. Cell Death Differ 18: 1271–1278. 21597463 10.1038/cdd.2011.59 PMC3172100

[46] Watanabe N, Lam E (2009) Bax Inhibitor-1, a conserved cell death suppressor, is a key molecular switch downstream from a variety of biotic and abiotic stress signals in plants. Int. J. Mol. Sci 10: 3149–3167. 19742129 10.3390/ijms10073149 PMC2738916

[47] Chen XH, Yu H, Deng HJ, Chen JX, MI HB, et al (2013) Cucumber BAX inhibitor-1, a conserved cell death suppressor and a negative programmed cell death regulator under cold stress. Biol Plantarum 57: 684–690.

[48] Cao ZH, Zhang SZ, Wang RK, Zhang RF, Hao YJ (2013) Genome wide analysis of the apple MYB transcription factor family allows the identification of MdoMYB121 gene conferring abiotic stress tolerance in plants. PLOS ONE 8: e69955. 23950843 10.1371/journal.pone.0069955 PMC3735319

[49] Reyes JL, Chua NH (2007) ABA induction of miR159 controls transcript levels of two MYB factors during *Arabidopsis* seed germination. Plant J 49: 592–606. 17217461 10.1111/j.1365-313X.2006.02980.x

[50] Seo PJ, Xiang F, Qiao M, Park JY, Lee YN, et al (2009) The MYB96 transcription factor mediates abscisic acid signaling during drought stress response in *Arabidopsis* . Plant Physiol 151: 275–289. 19625633 10.1104/pp.109.144220 PMC2735973

[51] Xie Z, Li D, Wang L, Sack FD, Grotewold E (2010) Role of the stomatal development regulators FLP/MYB88 in abiotic stress response. Plant J 64: 731–739. 21105921 10.1111/j.1365-313X.2010.04364.x

[52] Zhang L, Zhao G, Xia C, Jia J, Liu X, et al (2012) A wheat R2R3-MYB gene, TaMYB30-B, improves drought stress tolerance in transgenic *Arabidopsis* . J Exp Bot 63: 5873–5885. 23048128 10.1093/jxb/ers237

[53] Rushton PJ, Somssich IE, Ringler P, Shen QJ (2010) WRKY transcription factors. Trends Plant Sci 15: 247–258. 20304701 10.1016/j.tplants.2010.02.006

[54] Niu CF, Wei W, Zhou QY, Tian AG, Hao YJ, et al (2012) Wheat WRKY genes TaWRKY2 and TaWRKY19 regulate abiotic stress tolerance in transgenic *Arabidopsis* plants. Plant Cell Environ 35: 1156–1170. 22220579 10.1111/j.1365-3040.2012.02480.x

[55] Zhu X, Liu S, Meng C, Qin L, Kong L, et al (2013) WRKY transcription factors in wheat and their induction by biotic and abiotic stress. Plant Mol Biol Rep 31: 1053–1067.

[56] Arora R, Agarwal P, Ray S, Singh AK, Singh VP, et al (2007) MADS-box gene family in rice: genome-wide identification, organization and expression profiling during reproductive development and stress. BMC Genomics 8: 242. 17640358 10.1186/1471-2164-8-242 PMC1947970

[57] Puig J, Meynard D, Khong GN, Pauluzzi G, Guiderdoni E, et al (2013) Analysis of the expression of the AGL17-like clade of MADS-box transcription factors in rice. Gene Expr Patterns 13: 160–170. 23466806 10.1016/j.gep.2013.02.004

[58] Zhu JK (2002) Salt and drought stress signal transduction in plants. Annu Rev Plant Biol 53: 247–273. 12221975 10.1146/annurev.arplant.53.091401.143329 PMC3128348

[59] Wan D, Li R, Zou B, Zhang X, Cong J, et al (2012) Calmodulin-binding protein CBP60g is a positive regulator of both disease resistance and drought tolerance in *Arabidopsis* . Plant Cell Rep 31: 1269–1281. 22466450 10.1007/s00299-012-1247-7

[60] Ranty B, Aldon D, Galaud JP (2006) Plant calmodulins and calmodulin-related proteins: multifaceted relays to decode calcium signals. Plant Signal Behav 1: 96–104. 19521489 10.4161/psb.1.3.2998 PMC2635005

[61] Morris PC (2001) MAP kinase signal transduction pathways in plants. New Phytol 151: 67–89. 10.1046/j.1469-8137.2001.00167.x 33873387

[62] Singh A, Kanwar P, Pandey A, Tyagi AK, Sopory SK, et al (2013) Comprehensive genomic analysis and expression profiling of phospholipase C gene family during abiotic stresses and development in rice. PLOS ONE 8: e62494. 23638098 10.1371/journal.pone.0062494 PMC3640072

[63] Triantaphylidès C, Havaux M (2009) Singlet oxygen in plants: production, detoxification and signaling. Cell 14: 1360–1385. 10.1016/j.tplants.2009.01.008 19303348

[64] Kohli A, Sreenivasulu N, Lakshmanan P, Kuman PP (2013) The phytohormone crosstalk paradigm takes center stage in understanding how plants respond to abiotic stresses. Plant Cell Rep 32: 945–957. 23749097 10.1007/s00299-013-1461-y

[65] Peleg Z, Blumwald E (2011) Hormone balance and abiotic stress tolerance in crop plants. Curr Opin Plant Biol 14: 290–295. 21377404 10.1016/j.pbi.2011.02.001

[66] Santner A, Calderon-Villalobos LIA, Estelle M (2009) Plant hormones are versatile chemical regulators of plant growth. Nature Chem Biol 5: 301–307. 19377456 10.1038/nchembio.165

[67] Santino A, Taurino M, De Domenico S, Bonsegna S, Poltronieri P, et al (2013) Jasmonate signaling in plant development and defense response to multiple (a)biotic stresses. Plant Cell Rep 32: 1085–1098. 23584548 10.1007/s00299-013-1441-2

[68] Bleecker AB (2000) Ethylene: A gaseous signal molecule in plants. Annu Rev Cell Dev Biol 16: 1–18. 11031228 10.1146/annurev.cellbio.16.1.1

[69] Licausi F, Ohme-Takagi M, Perata P (2013) APETALA2/Ethylene responsive factor (AP2/ERF) transcription factors: mediators of stress responses and developmental programs. New Phytol 199: 639–649. 24010138 10.1111/nph.12291

[70] Teale WD, Paponov IV, Palme K (2006) Auxin in action: signaling, transport and the control of plant growth and development. Nature Rev 7: 847–859. 10.1038/nrm2020 16990790

[71] Pérez AC, Goossens A (2013) Jasmonate signaling: a copycat of auxin signaling? Environ 36: 2071–2084. 10.1111/pce.12121 23611666

[72] Boatwright J, Pajerowska-Mukhtar K (2013) Salicylic acid: an old hormone up to new tricks. Mol Plant Pathol 14: 623–634. 23621321 10.1111/mpp.12035 PMC6638680

[73] Cutler SR, Rodriguez PL, Finkelstein RR, Abrams SR (2010) Abscisic acid: emergence of a core signaling network. Annu Rev Plant Biol 61: 651–679. 20192755 10.1146/annurev-arplant-042809-112122

[74] Davière JM, Achard P (2013) Gibberellin signaling in plants. Dev 140: 1147–1151. 10.1242/dev.087650 23444347

[75] Richards DE, King KE, Ait-ali T, Harberd NP (2001) How gibberellin regulates plant growth and development: a molecular genetic analysis of gibberellin signaling. Annu Rev Plant Physiol 52: 67–88. 10.1146/annurev.arplant.52.1.67 11337392

[76] Harb A, Krishnan A, Ambavaram MMR, Pereira A (2010) Molecular and physiological analysis of drought stress in Arabidopsis reveals early responses leading to acclimation in plant growth. Plant Phys 154: 1254–1271. 10.1104/pp.110.161752 PMC2971604 20807999

[77] Damarajua S, Schledeb S, Eckhardta U, Loksteinb H (2011) GrimmaB (2011) Functions of the water soluble chlorophyll-binding protein in plants. J Plant Phys 168: 1444–1451. 10.1016/j.jplph.2011.02.007 21481489

[78] Gorantla M, Babu PR, Reddy Lachagari VB, Reddy AMM, Wusirika R, et al (2007) Identification of stress-responsive genes in an indica rice (*Oryza sativa* L.) using ESTs generated from drought-stressed seedlings. J Exp Bot 58: 253–265. 17132712 10.1093/jxb/erl213

[79] Guha A, Sengupta D, Reddy AR (2013) Polyphasic chlorophyll a fluorescence kinetics and leaf protein analyses to track dynamics of photosynthetic performance in mulberry during progressive drought. J Photochem Photobiol B 119: 71–83. 23357190 10.1016/j.jphotobiol.2012.12.006

[80] Kawasaki S, Miyake C, Kohchi T, Fujii S, Uchida M, et al (2000) Responses of wild watermelon to drought stress: accumulation of an ArgE homologue and citrulline in leaves during water deficits. Plant Cell Physiol 41: 864–873. 10965943 10.1093/pcp/pcd005

[81] Kusvuran S, Dasgan HY, Abak K (2013) Citrulline is an important biochemical indicator in tolerance to saline and drought stresses in melon. Scientific World J. 2013 ID:253414. 10.1155/2013/253414 PMC3864137 24363615

[82] Dasgan HY, KusvuranS, Abak K, Leport L, Larher F, et al (2009) The relationship between citrulline accumulation and salt tolerance during the vegetative growth of melon (*Cucumis melo* L.). Plant Soil Environ 55: 51–57.

