# Supplementary figures and images for: Analysis of the Citrullus colocynthis Transcriptome during Water Deficit Stress
Source: PLoS One. 2014 Aug 13;9(8):e104657. doi: 10.1371/journal.pone.0104657 (PMC4132101; doi:10.1371/journal.pone.0104657)

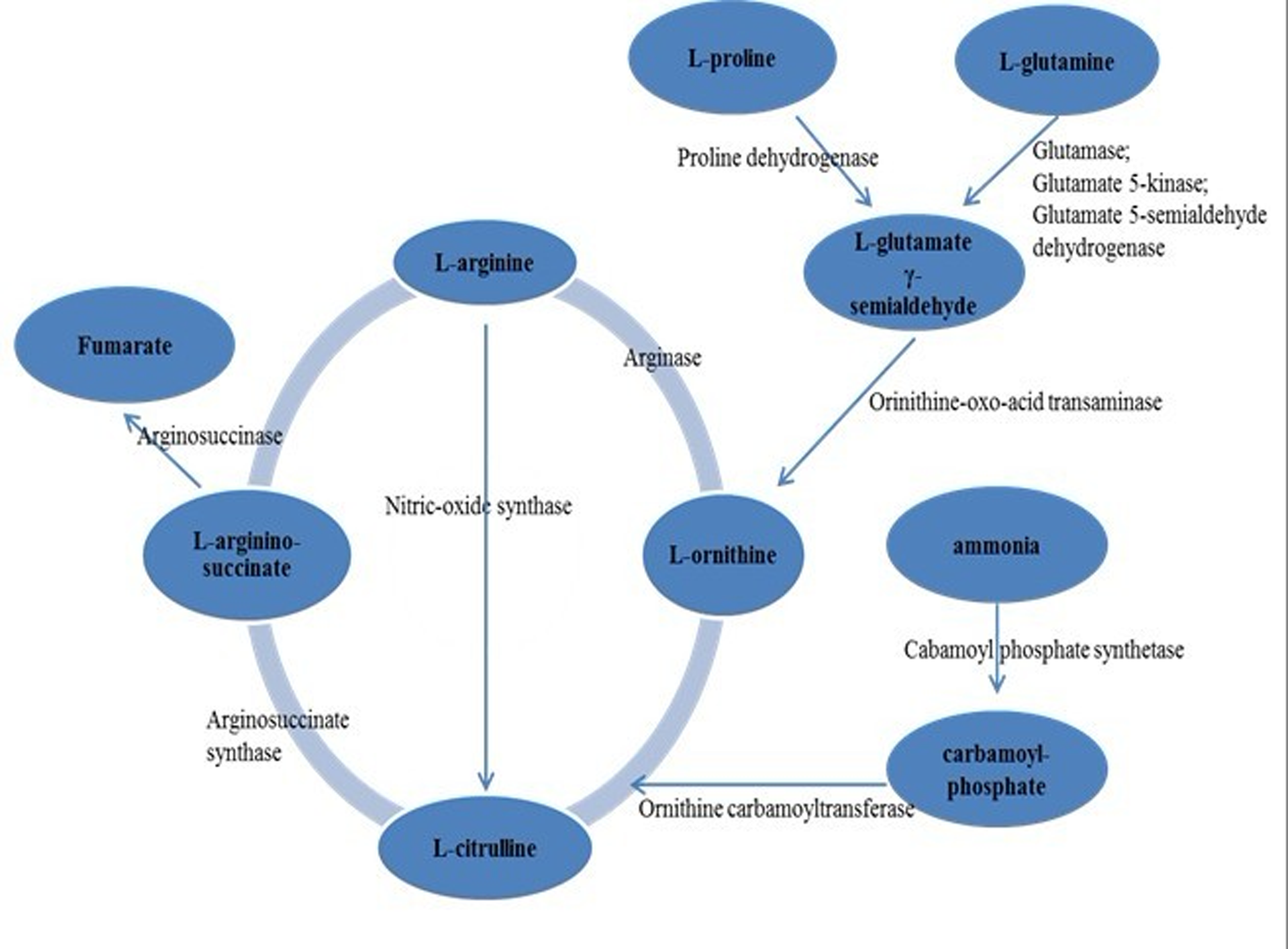

Supplement: Figure S1 — Citrulline metabolism pathway in C. colocynthis . (TIF) [file pone.0104657.s001.tif]
