# Supplementary material for: Analysis of the Citrullus colocynthis Transcriptome during Water Deficit Stress
Source: PLoS One. 2014 Aug 13;9(8):e104657. doi: 10.1371/journal.pone.0104657 (PMC4132101; doi:10.1371/journal.pone.0104657)
Supplement: Table S1 — qRT-PCR primer sequence information. (DOCX) [file pone.0104657.s002.docx]

**Table S1 qRT-PCR primer information**

| **Primer Name** | **Sequence (5' to 3')** | **length** |
| --- | --- | --- |
| Comp5873-F | TAAGCCGGAAATTGGTGATCAGTT | 24 |
| Comp5873-R | TTCTTCTATTGCTGCCTCTTCCCC | 24 |
| Comp862-F | AATGCTGCTTTGTTGCCTTCTGATC | 25 |
| Comp862-R | CCTCCTTCTTTCCTGTCTCATGCTCT | 26 |
| Comp1108-F | TCTTCGCGTATCCAAAAACAACATTA | 26 |
| Comp1108-R | CCAGCCAGACTCGCCCAATC | 20 |
| Comp10156-F | GAAGCCTCTAAGCACGTCGAAAGTT | 25 |
| Comp10156-R | ACCGAGACTACAACCGCCTTACATAC | 26 |
| Comp6528-F | ACGGAAGTCGAGAAAGATACGGATT | 25 |
| Comp6528-R | GCGTATGTTGGGTGAAATGGCA | 22 |
| Comp7317-F | TCCTCCTCACCATCCTCATCGTCT | 24 |
| Comp7317-R | GGTTGCGAGCGGAGACAGTGAG | 22 |
| Comp20554-F | CCCCCCGATTCTGCCGAC | 18 |
| Comp20554-R | ACCGCCTACTAAACTATCCATCCACTC | 27 |
| Comp3048-F | CCTTTTACCAGAGACTTTTCCCCAAT | 26 |
| Comp3048-R | CGTCCCTCTGTTCACCGGTTTC | 22 |
| Comp14675-F | TCAAACCCAGACCCCTCAAGAAAAC | 25 |
| Comp14675-R | GCGCTTGGATTGACATGCACC | 21 |
| Comp13927-F | ATCCCTTCATCCCCATTTTCCCTCT | 25 |
| Comp13927-R | CCAAAGCCGGGTATGTCGTCAAATC | 25 |
| Comp2553-F | CCCGAATTAATACGAAGCCTAGAAGA | 26 |
| Comp2553-R | CACTGATGCCATTTCTTTTTCTCTGTT | 27 |
| Comp372-F | CAGCGGGTTTATCTCACTCTTGGTT | 25 |
| Comp372-R | TTCAAAAAGAGCAGCCCCTAATAAAAT | 27 |
| Comp8117-F | AAGGAATTTGGGGATGGTTACAGAG | 25 |
| Comp8117-R | GGTTCTCTCCTTCCTTCCATTTCCA | 25 |
| Comp10586-F | AGAAAGGAACGCCATGGACTGAAGA | 25 |
| Comp10586-R | TTCTGGGCATGACTGGCTACCTGAG | 25 |
| Comp19751-F | AATGGAGGAAATATGGTCAAAAGGTG | 26 |
| Comp19751-R | ACCTTTCTCTTCACCGGACAGCTAG | 25 |
| Comp6823-F | GCTCAAGCATCAGTTCCACAAAATA | 25 |
| Comp6823-R | TGCTTCACAACGACATAACCTTCTT | 25 |
